# Cumulus Cells Block Oocyte Meiotic Resumption via Gap Junctions in Cumulus Oocyte Complexes Subjected to DNA Double-Strand Breaks

**DOI:** 10.1371/journal.pone.0143223

**Published:** 2015-11-17

**Authors:** Ming-Hong Sun, Jie Zheng, Feng-Yun Xie, Wei Shen, Shen Yin, Jun-Yu Ma

**Affiliations:** 1 College of Animal Science and Technology, Qingdao Agricultural University, Qingdao, Shandong, China; 2 Key Laboratory of Animal Reproduction and Germplasm Enhancement in Universities of Shandong, Qingdao Agricultural University, Qingdao, Shandong, China; 3 Institute of Reproductive Science, Key Laboratory of Animal Reproduction and Germplasm Enhancement in Universities of Shandong, College of Animal Science and Technology, Qingdao Agricultural University, Qingdao, Shandong, China; Institute of Zoology, Chinese Academy of Sciences, CHINA

## Abstract

During mammalian oocyte growth, genomic DNA may accumulate DNA double-strand breaks (DSBs) induced by factors such as reactive oxygen species. Recent evidence demonstrated that slight DSBs do not activate DNA damage checkpoint proteins in denuded oocytes. These oocytes, even with DNA DSBs, can resume meiosis and progress to metaphase of meiosis II. Meiotic resumption in oocytes is also controlled by the surrounding cumulus cells; accordingly, we analyzed whether cumulus-cell enclosed oocytes (CEOs) with DNA damage are able to resume meiosis. Compared with DNA-damaged denuded oocytes, we found that meiotic resumption rates of CEOs significantly decreased. To assess the mechanism by which cumulus cells block meiotic resumption in CEOs with DNA DSBs, we treated the cumulus oocyte complex with the gap junction inhibitor carbenoxolone and found that carbenoxolone can rescue the block in CEO meiosis induced by DNA DSBs. Since cumulus cell-synthesized cAMPs can pass through the gap junctions between oocyte and cumulus cell to block oocyte meiosis, we measured the expression levels of adenylate cyclase 1 (*Adcy1*) in cumulus cells, and G-protein coupled receptor 3 (*Gpr3*) and phosphodiesterase 3A (*Pde3a*) in oocytes, and found that the mRNA expression level of *Adcy1* increased significantly in DNA-damaged cumulus cells. In conclusion, our results indicate that DNA DSBs promote cAMP synthesis in cumulus cells, and cumulus cAMPs can inhibit meiotic resumption of CEOs through gap junctions.

## Introduction

Although mammalian oocytes initiate meiosis at the fetus stage, nearly all oocytes become arrested at prophase of meiosis I a few days before or after birth[1]. Oocyte arrest can last for many weeks in mice and decades in humans. Upon stimulation by follicle-stimulating hormone, follicles are activated and oocytes begin to grow. All growing oocytes remain arrested at prophase I and do not resume meiosis until oocyte growth is complete and corresponding follicles are stimulated by luteinizing hormone[2]. Similar to somatic cells, the genomic DNA of oocyte is subject to double-strand breaks (DSBs) by factors such as reactive oxygen species, drugs, or radiation. In somatic cells, to maintain genomic DNA integrity, DNA repair proteins can sense DNA damage events, such as DSBs, and transmit signals to checkpoint proteins to arrest the cell cycle[3, 4]; additional DNA damage repair proteins are then recruited to the site of damage to repair DNA[5–7]. In comparison to somatic cells, oocytes are less sensitive to DNA DSBs. When the genomic DNA of denuded oocytes has slight double-strand breaks, oocytes do not activate DNA damage checkpoint proteins, and subsequently can resume meiosis, which was identified by germinal vesicle breakdown(GVBD), with damaged genome[8, 9].

Unlike denuded oocytes, mature oocytes *in vivo* are surrounded by multilayered cumulus cells. The cumulus cells and oocyte form a closely connected complex called the cumulus oocyte complex (COC); the COC is connected by gap junctions between cumulus cells, as well as between cumulus cells and oocytes [10]. Evidence shows that the second messenger cAMP is transferred from cumulus cells into oocytes through gap junctions [11, 12], and that elevated cAMP in oocytes maintains oocyte meiotic arrest [13]. In addition to passive transfer of cAMP from cumulus cells to oocyte, the oocyte itself can also regulate its own cellular cAMP level. Oocyte G-protein coupled receptor 3 (Gpr3) can maintain oocyte meiotic arrest by regulating cAMP levels in oocytes[1, 14]. Conversely, oocyte phosphodiesterase 3A (Pde3a) is activated following luteinizing hormone stimulation to hydrolyze cAMPs in oocytes and promote meiotic resumption[1, 15].

To investigate the effects of DNA DSBs on CEO meiotic resumption, and to assess whether cumulus cells can prevent meiotic resumption in DNA-damaged oocytes, we induced DNA DSBs in COCs using the drug Zeiocin and measured the maturation rates of CEOs with DNA DSBs. We also assessed communication between DNA-damaged oocytes and cumulus cells. Finally, we measured the mRNA levels of adenylate cyclase 1 (*Adcy1*) [16]in DNA damaged cumulus cells and *Gpr3*and *Pde3a*in oocytes.

## Materials and Methods

### Ethics Statement

The ICR outbred mice were purchased from the laboratory animal breeding center of Wenbo. Cervical dislocation method was used for mouse euthanasia. All mouse manipulations were performed with the approval of the Animal Care and Ethics Committee of Qingdao Agricultural University.

### Isolation and culturing of COCs and denuded oocytes

COCs were isolated from ovaries of female ICR outbred mice 48 hours post-PMSG injection. Denuded oocytes were isolated from ovaries from female ICR mice without PMSG injection. All oocytes manipulations were performed in manipulation medium M2 (Sigma) containing 2.5 μM milrinone (Amyjet) to prevent GVBD. Both COCs and denuded oocytes were matured *in vitro* in MEMα medium (Gibco) containing 10% FBS, 0.5 μ0% F FSH, 0.5 μFSH, LH, 10 ng/mL EGF, and 1% Penicillin-Streptomycin Solution (HyClone) at 37°C with 5% CO_2_.

#### Drugs used for oocyte treatment

In our experiment, we treated oocytes (denuded oocytes or COCs) with two drugs: the DNA DSB-inducing antibiotic, Zeiocin (Invitrogen), and the gap junction inhibitor, carbenoxolone (CBX, Sigma). Denuded oocytes or COCs were treated with 200 μg/mL Zeiocin for 1 hour in oocyte maturation medium with milrinone to induce DNA DSBs. To inhibit gap junctions, COCs or denuded oocytes were cultured in maturation medium containing 200 μM CBX.

#### Immunostaining

Both COCs and cumulus cells and zona pellucida removed oocytes were used for immunostaining. The cumulus cells of COC were removed by vibrating for 10 minutes. The zona pellucida was removed by acidic Tyrode solution treating. COCs and Oocytes were fixed in 4% paraformaldehyde for 40 minutes, and then permeabilized in PBS with 0.1% Triton X-100 for 20 minutes. Next, oocytes were blocked in PBS containing 1% BSA for 1 hour and incubated with γH2A.X antibody (Bioworld) and/or cAMP antibody (AM01, Santa Cruz Biotechnology) at 4°C overnight. After 3 washes with PBST, oocytes were incubated with secondary antibody (Anti-Rabbit IgG FITC conjugate, Sigma; Cy3 conjugated goat anti mouse IgG antibody, Beyotime) and washed 5 times with PBST. DNA was stained with Hoechst stain and the oocytes were mounted on slides. We used a confocal laser-scanning microscope to observe the oocytes.

#### Quantitative real time-PCR

To isolate cumulus cells from COCs, COCs in M2 medium were vibrated using a vortex oscillator for 5–10 minutes. The cumulus cells and oocytes were collected for RNA extraction. The mRNAs of denuded oocytes, oocytes from COCs, or cumulus cells were isolated using the RNeasy Micro Kit (Qiagen) and reverse transcribed to cDNAs by TransScript One-Step gDNA Removal and cDNA Synthesis SuperMix (TransGene). These cDNAs were used as templates for quantitative real time-PCR (qRT-PCR); qRT-PCR was performed on the Lightcycler 480 II (Roche) platform using the Lightcycler 480 SYBR Green I Master kit (Roche). The primers used for qRT-PCR were as follows: *Pde3a*-F: AATGGGACCACAAGAGAGGG, *Pde3a*-R: TTCACTCTGGGCTTGTGGAT; *Gpr3*-F: TATCCACTCTCCAAGAACCATCTGG, *Gpr3*-R: GGAATTAAGCCCTGGTGGACCTAAC; *Adcy1*-F: GTGGTGGCTGCCTCGCACTT, *Adcy1*-R: AGCAGGGCATTGGCACCGAG; *Gapdh*-F: GTCATTGAGAGCAATGCCAG, *Gapdh*-R: GTGTTCCTACCCCCAATGTG.

### Statistic analysis

Student’s *t*-test was used to assess statistical significance. Data are represented as means and standard errors. The statistical software R was used to calculate the statistic values.

## Results and Discussion

### CEOs are more sensitive to DNA DSBs than denuded oocytes

As shown in previous studies, denuded oocytes are subject to GVBD when their DNA is slight double-strand broken [8, 9, 17]. In denuded oocytes, we found that GVBD occurred in 76.4% of normal oocytes (N = 80) but only in 31.6% of oocytes with Zeiocin-induced DSBs(N = 75) after 4 hours in culture. The GVBD rates of normal and DSB denuded oocytes increased to 83.9% and 59.0%, respectively, when the oocytes were cultured for 15 hours.

**Fig 1:**
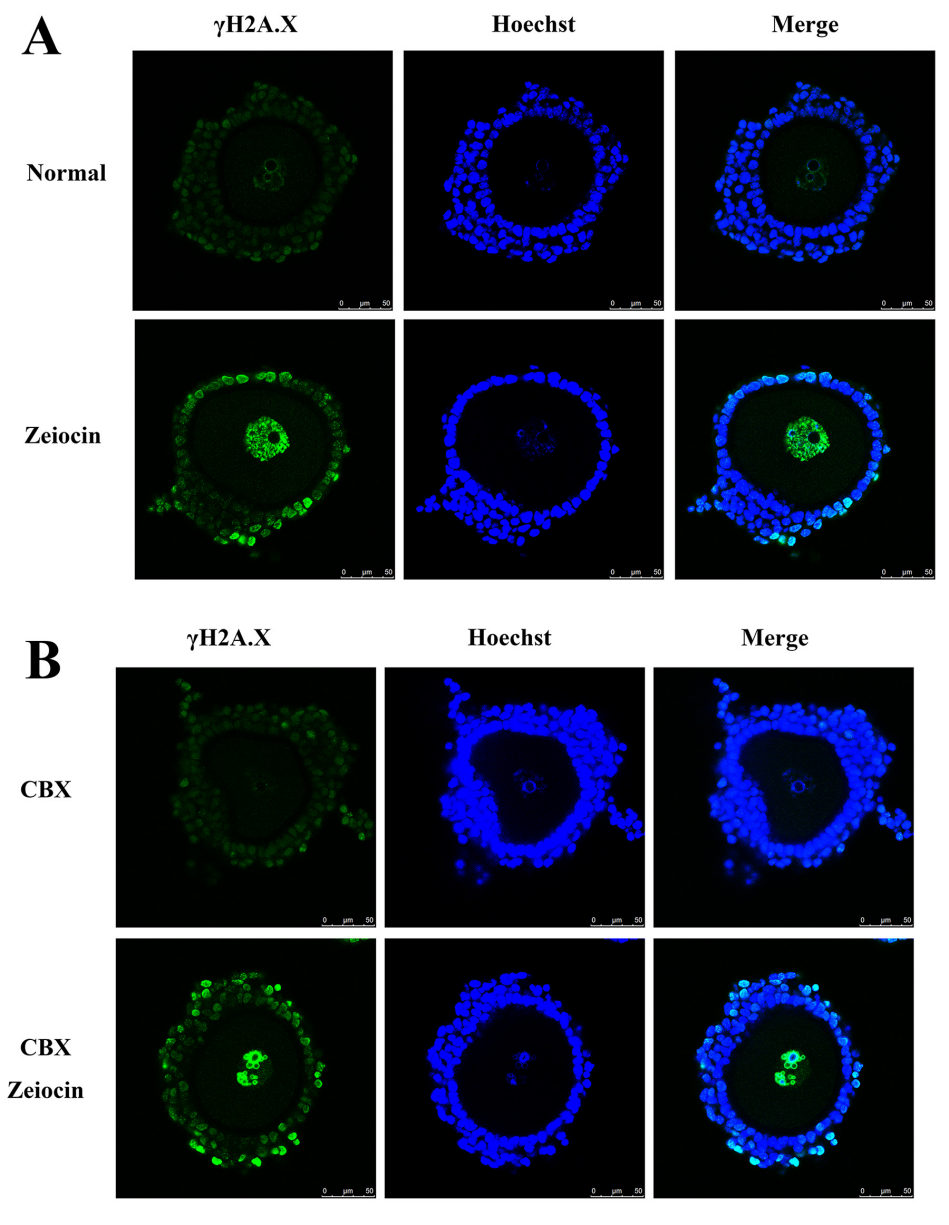
DNA double-stand breaks in normal, Zeiocin-treated, carbenoxolone (CBX)- treated and Zeiocin/CBX-double treated COCs. (A) Normal and zeiocin-treated COCs. (B) CBX-treated normal and DNA-damaged oocytes. γH2A.X, serine 139 phosphorylated histone H2A.X, a marker of DNA double-strand breaks.

**Fig 2:**
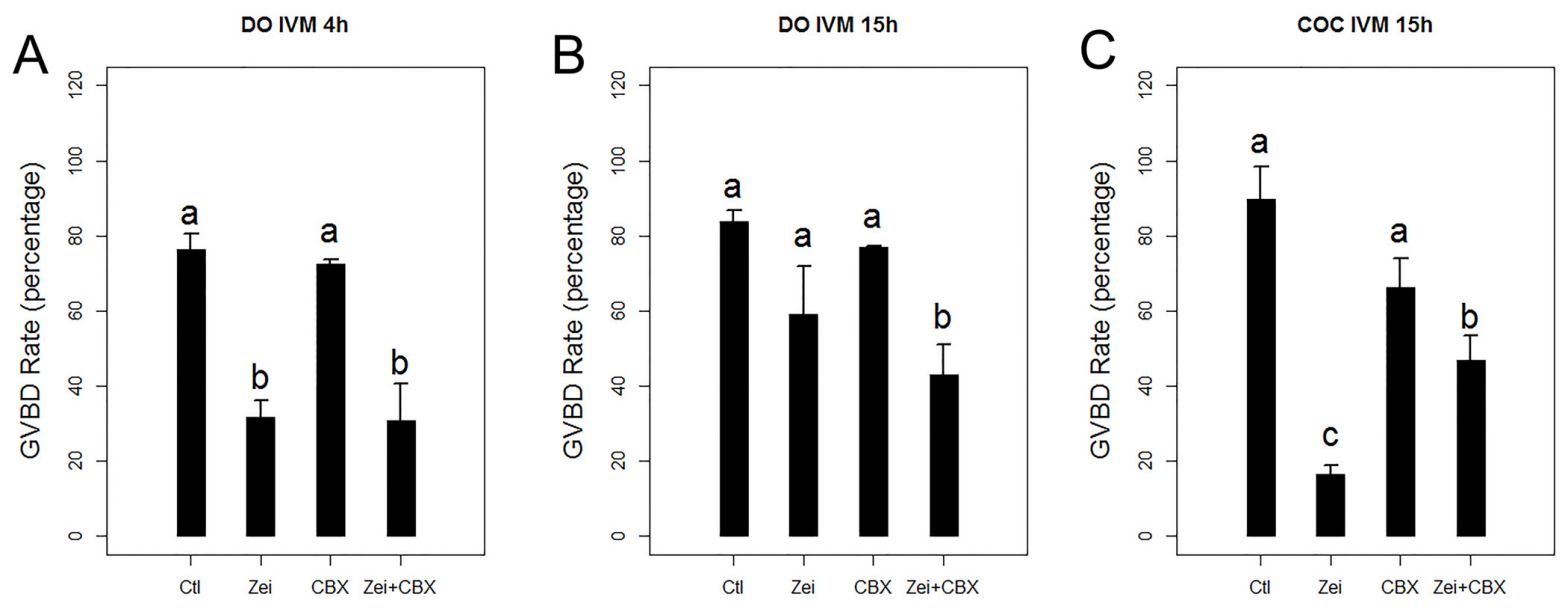
Germinal vesicle breakdown (GVBD) rates of denuded oocytes and cumulus enclosed oocytes (CEOs). (A) GVBD rates of denuded oocytes after 4 hours *in vitro* maturation (IVM). (B) GVBD rates of denuded oocytes after 15 hours IVM. (C) GVBD rates of CEOs after 4 hours IVM. COC, cumulus enclosed oocyte. Ctl, control or normal group; Zei, Zeiocin-treated group; CBX, carbenoxolone-treated group; Zei+CBX, Zeiocin/CBX-double treated group.

To validate Zeiocin induction of DSBs, DSBs in COCs were identified by immunostaining of serine 139 phosphorylated histone H2A.X (γH2A.X), an early marker of DNA DSBs[18]. When COCs were treated with Zeiocin, both oocytes and cumulus cells exhibited DNA DSBs (Fig 1A). Compared with denuded oocytes, when COCs were treated with Zeiocin, the GVBD rate of normal CEOs was 89.8% (N = 73) and only 16.4% for DSB CEOs (N = 84, p < 0.01), significantly less than that of denuded oocytes, after 15 hours in culture (Fig 2).

The above results indicated although oocytes themselves are less sensitive to DNA DSBs, on the normal Physiological conditions, cumulus cells can regulate the cell cycle of oocytes when the COCs were DNA damaged.

### The DNA DSB-induced block in meiosis in CEOs can be rescued by the gap junction inhibitor CBX

To analyze whether the decrease in GVBD rates of CEOs with DNA DSBs was induced by cumulus cells via gap junctions, we used CBX to block the gap junctions in COCs (Fig 1). When denuded oocytes were treated with CBX, the GVBD rates of CBX-treated normal oocytes (72.5%, N = 91) and DNA DSB oocytes (30.7%, N = 69) were not significantly different than the oocytes without CBX treatment after 4 hours *in vitro* maturation. The GVBD rates of CBX-treated normal and DNA DSB denuded oocytes increased to 76.9% and 42.9%, respectively, after 15 hours in culture.

For normal COCs, when treated with CBX, the GVBD rate of CEOs decreased, but not significantly (66.1%, N = 68). For the DNA DSB COCs, CBX-treatment increased the GVBD rate of CEOs significantly (46.9%, N = 68, Fig 2). These results indicated the DNA damaged cumulus cells suppress the GVBD of CEOs through gap junctions.

### DNA DSBs increase *Adcy1* expression in cumulus cells and induced the enrichment of cAMPs at the zona pellucida and nucleus of oocytes

**Fig 3:**
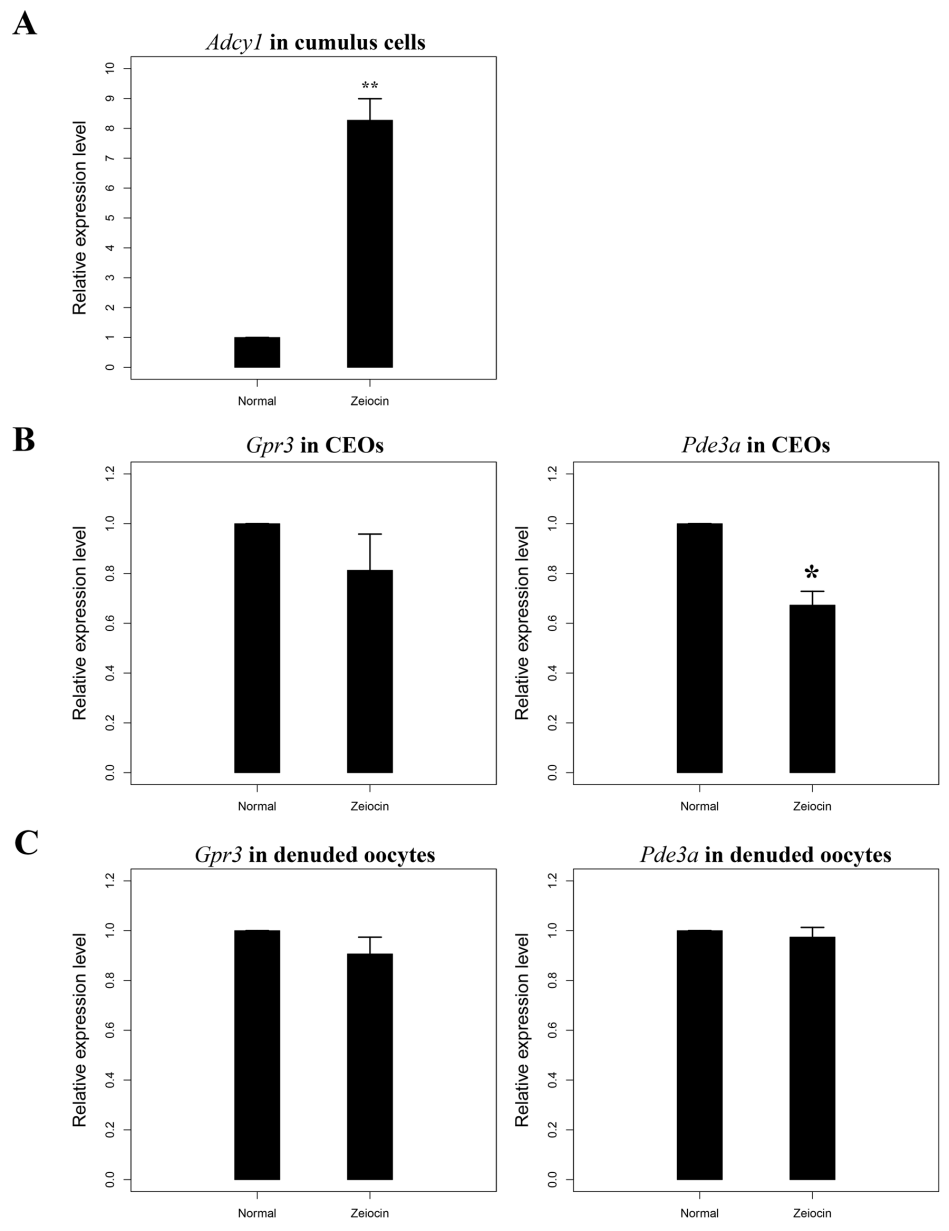
DNA double-strand breaks (DSBs) up-regulate *Adcy1* in cumulus cells and down-regulate *Pde3a* in cumulus-enclosed oocytes. (A) *Adcy1* is up-regulated in the cumulus cells from cumulus oocyte complexes (COCs)with DNA DSBs.(B) The expression of *Gpr3* and *Pde3a* in oocytes from normal and DNA DSB COCs. (C)The expression of *Gpr3* and *Pde3a* in normal and DNA DSB denuded oocytes. **, highly significant difference, p < 0.01; *, significant difference, 0.01 < p < 0.05.

cAMP is one of the most important second messengers synthesized by cumulus cells and can control oocyte meiotic progression. As the mRNA level of *Acdy1* in oocyte is very low (Ct value of *Gapdh* is about 21–24, but the Ct of *Acdy1* is 35–40, data not shown), the cAMP in oocyte may mainly rely on the cumulus cells. To assess whether cumulus cells inhibit GVBD in oocytes via cAMP synthesis from COCs containing DNA DSBs, we measured mRNA levels of the *Adcy1* gene in cumulus cells. Consistent with our expectations, the qRT-PCR results showed that the *Adcy1* transcription level increased significantly in cumulus cells with DNA DSBs (Fig 3). Our results indicate that DNA DSBs up-regulate the expression of *Adcy1* in cumulus cells, which will subsequently increase cAMP levels in cumulus cells.

**Fig 4:**
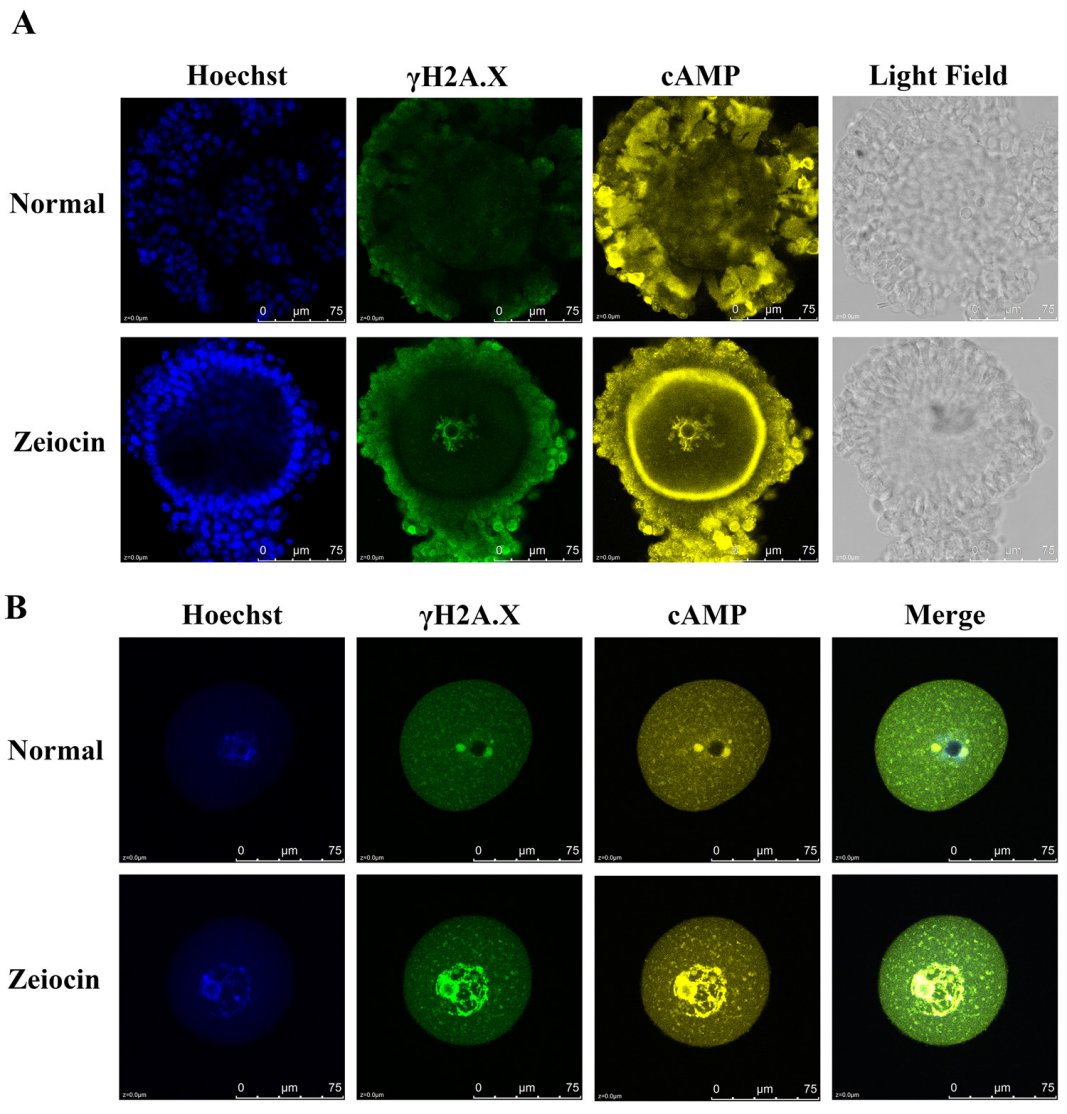
Immunostaining of cAMP in DSB COCs. (A) cAMP enriched in the zona pellucida of DSB COCs. (B) Cumulus cells of COCs were removed by vibrating, and the zona pellucida was removed by acidic Tyrode solution. Compared with normal group, the nucleus of DSB oocytes enriched with cAMP. Blue, Hoechst; Green, γH2A.X; yellow, cAMP. DSB, DNA double-strand breaks.

Previous studies showed that cAMPs are transferred from cumulus cells into oocytes through gap junctions [12], and that increased oocyte intracellular cAMPs inhibit meiotic resumption [1]. We immunostained cAMP in DSB COCs and the cumulus cells and zona pellucida removed oocytes (Fig 4), our results showed cAMP enriched in the zona pellucida and nucleus of DSB oocytes. These results indicated the cumulus cells can control oocyte meiosis by producing and transporting cAMPs when the follicle was affected by DNA damage.

### DNA DSBs decrease *Pde3a* but not *Gpr3* expression in CEOs

During mouse oocyte growth, Gpr3 maintains cAMP levels in oocytes and is important for oocyte meiotic arrest [19, 20]. Pde3a, which can hydrolyze cAMPs, is expressed primarily in oocytes, but not in cumulus cells [21]. To assess whether DNA DSBs affect the expression of *Gpr3* and *Pde3a* in oocytes, we measured their mRNA levels in denuded oocytes and CEOs; mRNA levels of *Gpr3* in denuded oocytes and CEOs showed no significant changes when oocytes contained DNA DSBs. The expression of *Pde3a* was slightly decreased in DNA-damaged CEOs but there was no significant change in *Pde3a* mRNA levels between normal and denuded oocytes with DNA DSBs (Fig 3). The down-regulation of *Pde3a* in DNA DSB CEOs likely decreases the hydrolysis of cAMP and further suppresses meiotic resumption of CEOs.

## Conclusions

**Fig 5:**
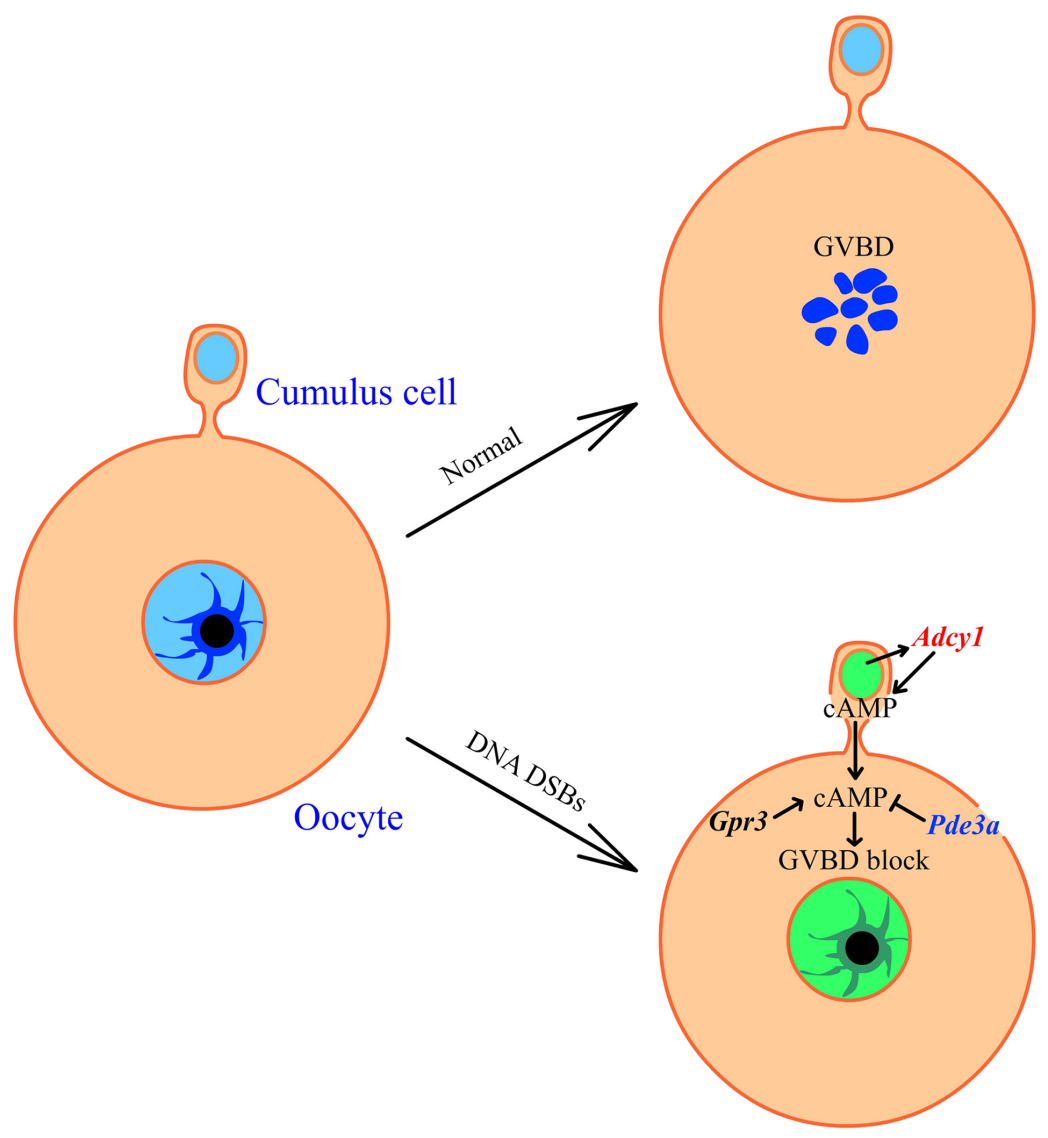
Model of cumulus cell regulating oocyte meiosis when the COC DNA was DSB. The DNA DSBs (Green) increase the transcription of Adcy1 (red) and cAMP production in cumulus cells. cAMPs are transported from cumulus cells to oocytes through gap junctions. The Gpr3 maintains the cAMP level in oocytes and Pde3a hydrolyze cAMPs in oocytes. The transcription of *Gpr3* showed no significant change in DSB oocytes, where *Pde3a* (blue) is down regulated in DSB oocytes. Enrichment of cAMP in oocytes induce the oocyte GVBD block. DSB, DNA double-strand breaks; GVBD, germinal vesicle breakdown.

In conclusion, our study revealed that cumulus cells block the meiotic resumption of oocytes from COC containing DNA double-strand breaks by promoting cumulus cell cAMP synthesis. We also found that cumulus cells control oocyte meiosis through gap junctions and the cAMP enriched in the zona pellucida and nucleus of DSB cumulus enclosed oocytes. In addition, we demonstrated slight down-regulation of *Pde3a* in DNA-damaged CEOs. Finally, from our data we found a new mechanism about how ovaries prevent the DNA damaged oocytes from GVBD: when COCs were DNA DSB damaged, cumulus would transport more cAMPs to oocytes through the gap junctions between oocytes and cumulus cells, and the accumulation of cAMPs in oocytes blocked the GVBD of CEOs (Fig 5). Meanwhile, the expression of *Adcy1* is up-regulated in cumulus cells when the COC was DNA damaged, which will promote the cumulus cell produce more cAMP to block the GVBD of oocytes (Fig 5). Taken together, these results indicate that oocyte meiotic resumption is not only controlled by the oocyte itself but also by the surrounding cumulus cells when the COCs are damaged by external factors.

## References

[1] Sun QY, Miao YL, Schatten H. Towards a new understanding on the regulation of mammalian oocyte meiosis resumption. Cell Cycle. 2009;8(17):2741–7. Epub 2009/09/01. . 19717979 10.4161/cc.8.17.9471

[2] Mehlmann LM. Stops and starts in mammalian oocytes: recent advances in understanding the regulation of meiotic arrest and oocyte maturation. Reproduction. 2005;130(6):791–9. Epub 2005/12/03. 10.1530/rep.1.00793 . 16322539

[3] Sancar A, Lindsey-Boltz LA, Unsal-Kacmaz K, Linn S. Molecular mechanisms of mammalian DNA repair and the DNA damage checkpoints. Annual review of biochemistry. 2004;73:39–85. Epub 2004/06/11. 10.1146/annurev.biochem.73.011303.073723 . 15189136

[4] Sperka T, Wang J, Rudolph KL. DNA damage checkpoints in stem cells, ageing and cancer. Nature reviews Molecular cell biology. 2012;13(9):579–90. Epub 2012/08/24. 10.1038/nrm3420 . 22914294

[5] Abraham RT. Cell cycle checkpoint signaling through the ATM and ATR kinases. Genes & development. 2001;15(17):2177–96. Epub 2001/09/07. 10.1101/gad.914401 . 11544175

[6] Langerak P, Russell P. Regulatory networks integrating cell cycle control with DNA damage checkpoints and double-strand break repair. Philosophical transactions of the Royal Society of London Series B, Biological sciences. 2011;366(1584):3562–71. Epub 2011/11/16. 10.1098/rstb.2011.0070 22084383 PMC3203453

[7] Lord CJ, Ashworth A. The DNA damage response and cancer therapy. Nature. 2012;481(7381):287–94. Epub 2012/01/20. 10.1038/nature10760 . 22258607

[8] Ma JY, Ou Yang YC, Wang ZW, Wang ZB, Jiang ZZ, Luo SM, et al The effects of DNA double-strand breaks on mouse oocyte meiotic maturation. Cell Cycle. 2013;12(8):1233–41. Epub 2013/03/23. 10.4161/cc.24311 23518501 PMC3674088

[9] Marangos P, Carroll J. Oocytes progress beyond prophase in the presence of DNA damage. Current biology: CB. 2012;22(11):989–94. Epub 2012/05/15. 10.1016/j.cub.2012.03.063 . 22578416

[10] Kidder GM, Mhawi AA. Gap junctions and ovarian folliculogenesis. Reproduction. 2002;123(5):613–20. Epub 2002/05/15. . 12006089 10.1530/rep.0.1230613

[11] Webb RJ, Marshall F, Swann K, Carroll J. Follicle-stimulating hormone induces a gap junction-dependent dynamic change in [cAMP] and protein kinase a in mammalian oocytes. Developmental biology. 2002;246(2):441–54. Epub 2002/06/08. 10.1006/dbio.2002.0630 . 12051828

[12] Bornslaeger EA, Schultz RM. Regulation of mouse oocyte maturation: effect of elevating cumulus cell cAMP on oocyte cAMP levels. Biology of reproduction. 1985;33(3):698–704. Epub 1985/10/01. . 2996645 10.1095/biolreprod33.3.698

[13] Bornslaeger EA, Mattei P, Schultz RM. Involvement of cAMP-dependent protein kinase and protein phosphorylation in regulation of mouse oocyte maturation. Developmental biology. 1986;114(2):453–62. Epub 1986/04/01. . 2420661 10.1016/0012-1606(86)90209-5

[14] Mehlmann LM, Saeki Y, Tanaka S, Brennan TJ, Evsikov AV, Pendola FL, et al The Gs-linked receptor GPR3 maintains meiotic arrest in mammalian oocytes. Science. 2004;306(5703):1947–50. Epub 2004/12/14. 10.1126/science.1103974 . 15591206

[15] Wigglesworth K, Lee KB, O'Brien MJ, Peng J, Matzuk MM, Eppig JJ. Bidirectional communication between oocytes and ovarian follicular somatic cells is required for meiotic arrest of mammalian oocytes. Proceedings of the National Academy of Sciences of the United States of America. 2013;110(39):E3723–9. Epub 2013/08/28. 10.1073/pnas.1314829110 23980176 PMC3785791

[16] Wang Y, Teng Z, Li G, Mu X, Wang Z, Feng L, et al Cyclic AMP in oocytes controls meiotic prophase I and primordial folliculogenesis in the perinatal mouse ovary. Development. 2015;142(2):343–51. Epub 2014/12/17. 10.1242/dev.112755 . 25503411

[17] Yuen WS, Merriman JA, O'Bryan MK, Jones KT. DNA double strand breaks but not interstrand crosslinks prevent progress through meiosis in fully grown mouse oocytes. PloS one. 2012;7(8):e43875 Epub 2012/08/29. 10.1371/journal.pone.0043875 22928046 PMC3425511

[18] Glover L, Horn D. Trypanosomal histone gammaH2A and the DNA damage response. Molecular and biochemical parasitology. 2012;183(1):78–83. Epub 2012/02/23. 10.1016/j.molbiopara.2012.01.008 22353557 PMC3334830

[19] Hinckley M, Vaccari S, Horner K, Chen R, Conti M. The G-protein-coupled receptors GPR3 and GPR12 are involved in cAMP signaling and maintenance of meiotic arrest in rodent oocytes. Developmental biology. 2005;287(2):249–61. Epub 2005/10/19. 10.1016/j.ydbio.2005.08.019 . 16229830

[20] Mehlmann LM. Oocyte-specific expression of Gpr3 is required for the maintenance of meiotic arrest in mouse oocytes. Developmental biology. 2005;288(2):397–404. Epub 2005/11/18. 10.1016/j.ydbio.2005.09.030 16289135 PMC1868506

[21] Shitsukawa K, Andersen CB, Richard FJ, Horner AK, Wiersma A, van Duin M, et al Cloning and characterization of the cyclic guanosine monophosphate-inhibited phosphodiesterase PDE3A expressed in mouse oocyte. Biology of reproduction. 2001;65(1):188–96. Epub 2001/06/23. . 11420239 10.1095/biolreprod65.1.188

